# Editorial: Advancements in HPV research: integrating diagnostics, vaccination, and women’s health

**DOI:** 10.3389/frhs.2025.1769805

**Published:** 2026-01-12

**Authors:** Varsetile Varster Nkwinika, Zeenat Ismail, Abdu Abdullahi Adamu, Oliver Ombeva Malande, Harris Onywera

**Affiliations:** 1 Department of Virology, Sefako Makgatho Health Sciences University, Pretoria, South Africa; 2 South African Vaccination and Immunisation Centre, Sefako Makgatho Health Sciences University, Pretoria, South Africa; 3 Department of Public Health Pharmacy and Management, Sefako Makgatho Health Sciences University, Pretoria, South Africa; 4 Regional Office for Africa, World Health Organization, Brazzaville, Democratic Republic of Congo; 5 Department of Child Health and Paediatrics, Moi University, Eldoret, Kenya; 6 Department of Paediatrics and Child Health, Makerere University, Kampala, Uganda; 7 The East African Centre for Vaccines & Immunization (ECAVI), Kampala, Uganda; 8 Institute of Infectious Disease and Molecular Medicine, University of Cape Town, Cape Town, South Africa; 9 Division of Medical Microbiology, Department of Pathology, Faculty of Health Sciences, University of Cape Town, Cape Town, South Africa

**Keywords:** cervical cancer prevention, HPV screening, HPV vaccination, human papillomavirus - HPV, implementation science

## 1 Introduction

Human papillomavirus (HPV) infection leading to cervical cancer remains one of the most urgent public health challenges worldwide. In 2022 alone, there were an estimated 662,301 new cervical cancer cases and 348,874 deaths globally, with the burden heavily affecting low- and middle-income countries (LMICs) where access to prevention, screening, and treatment remains limited (1, 2). Persistent infection with high-risk HPV genotypes, especially types 16 and 18, underpins nearly all cases of cervical cancer, causing over 300,000 deaths each year (3). Despite significant scientific advances in HPV diagnostics and vaccine development over the past two decades, translating these innovations into equitable healthcare practices remains a major global challenge (4). A clearer understanding of how best to deliver evidence-based interventions in real-world settings is crucial to bridging the gap between research and actual health outcomes, especially in low-resource settings where vaccination uptake, screening coverage, sociocultural barriers, and health system limitations continue to pose challenges.

In the current Frontiers thematic Research Topic titled “*Advancements in HPV Research: Integrating Diagnostics, Vaccination, and Women’s Health*”, this essential need is the central focus. By bringing together multidisciplinary studies covering HPV prevalence, innovative diagnostics, vaccination strategies, behavioural determinants, and implementation science, the collection offers up-to-date evidence on both scientific progress and real-world barriers across the continuum of HPV prevention and control. The five articles featured in this Research Topic provide evidence-based insights across the entire range of HPV prevention and control, including vaccine immunogenicity and optimisation, screening implementation and acceptability, uptake challenges, self-sampling strategies, and public perceptions of vaccination. Collectively, the contributions converge around three interconnected themes.

Optimising HPV screening implementation and acceptability
Understanding determinants of HPV vaccination and public perceptions
Strengthening evidence on vaccine effectiveness, age of administration, and dose strategies

### 1.1 Screening implementation and acceptability

A key question in the global effort to eliminate cervical cancer is how HPV screening can be effectively integrated into different health systems.

A qualitative study by Hahn et al. investigated the implications between locally tailored and centrally administered HPV screening strategies within an integrated healthcare network. Their qualitative findings underscore a recurrent theme across implementation research: sustainability depends not on technical capability alone, but on the alignment of screening processes with local workflows, stakeholder priorities, and institutional cultures. Their contribution highlights the importance of empowering frontline teams in adapting screening protocols to real-world conditions.

Complementing these findings, Yang et al. provide insights from a cross-sectional study assessing HPV self-sampling among women in Zhengzhou, China. Their work demonstrates robust acceptability of self-sampling and reinforces the value of community-based approaches, especially in contexts where stigma, cost, or access to facilities pose barriers to traditional clinician-collected samples. Together, these articles show that successful implementation depends on flexibility, community engagement, and sensitivity to local socioeconomic conditions.

### 1.2 Determinants of HPV vaccine uptake and public perception

HPV vaccination has proven highly effective in reducing cervical disease, yet uptake varies considerably, often reflecting underlying social and structural factors.

Mengistie et al. conducted an umbrella review synthesising the available evidence on determinants of vaccine uptake among women in Africa. Their analysis identifies persistent inequalities driven by misinformation, inadequate health education, limited health system capacity, and structural barriers such as distance and cost. The review emphasises that improving vaccination coverage requires multipronged approaches, policy alignment, community-based interventions, and targeted communication strategies.

The social dimension of vaccination is further examined by Matsangaise et al., who analysed vaccine sentiment on South African social media prior to the COVID-19 pandemic. Their study reveals widespread mistrust and misinformation, highlighting that digital platforms play a significant role in shaping public perceptions of vaccination. These findings reflect global concerns about the profound and often overlooked impact of online discourse on vaccine confidence.

### 1.3 Vaccine effectiveness, age at vaccination, and dose optimization

A final theme relates to the immunological and programme evidence informing HPV vaccine schedules.

Rostami Varnousfaderani et al. synthesise current evidence on vaccine effectiveness in relation to age of administration and number of doses. Their review highlights two critical points:

- vaccination before sexual debut offers the highest level of durable protection, and
- accumulating evidence supports the effectiveness of reduced-dose schedules, including single-dose strategies.

These findings have significant implications for resource-limited settings, where cost and logistical barriers can hinder the completion of multi-dose regimens.

## 2 Broader implications and future directions

Individually and collectively, the contributions in this research topic emphasise that scientific advances alone are insufficient to attain equitable HPV prevention. Effective integration demands attention to three overarching imperatives:

### 2.1 Linking diagnostics and vaccination in unified care pathways

Health systems should prioritise integrated models that bring screening, vaccination, and follow-up care into the same continuum to improve uptake and retention.

### 2.2 Addressing misinformation and strengthening public trust

As demonstrated by Matsangaise et al., communication challenges can undermine even the most robust scientific tools. Enhancing health literacy and combating misinformation must remain core components of national immunisation strategies.

### 2.3 Adapting interventions to local sociocultural and health system realities

There is no single universal approach. Implementation must consider context-specific barriers, resource availability, and cultural norms to achieve meaningful and sustainable progress.

While progress is evident, significant gaps persist, especially in strategies for engaging underserved populations, maintaining long-term vaccine confidence, and translating scientific evidence into national policy.

## 3 Conclusion

The collective findings of these studies highlight that integrating diagnostics, vaccination, and women's health through an implementation science lens is essential to accelerating regional and global efforts to eliminate HPV. By placing implementation strategies, vaccine uptake, communication dynamics, and immunogenicity within broader health system and societal contexts, this collection enhances both scientific understanding and practical measures. Continued interdisciplinary collaboration will be key to ensuring that innovations lead to equitable and measurable reductions in HPV-related disease burden.
